# Dynamic knee valgus alignment influences impact attenuation in the lower extremity during the deceleration phase of a single-leg landing

**DOI:** 10.1371/journal.pone.0179810

**Published:** 2017-06-20

**Authors:** Akihiro Tamura, Kiyokazu Akasaka, Takahiro Otsudo, Jyunya Shiozawa, Yuka Toda, Kaori Yamada

**Affiliations:** 1 Saitama Medical University Graduate School of Medicine, Moroyama, Saitama, Japan; 2 Saitama Medical University, School of Physical Therapy, Moroyama, Saitama, Japan; 3 Department of Rehabilitation, Zenshukai Hospital, Maebashi, Gunma, Japan; 4 Department of Rehabilitation, Yokohama Asahi Center General Hospital, Yokohama, Kanagawa, Japan; 5 Department of Rehabilitation, Kanetsu Hospital, Tsurugashima, Saitama, Japan; West Virginia University, UNITED STATES

## Abstract

Dynamic knee valgus during landings is associated with an increased risk of non-contact anterior cruciate ligament (ACL) injury. In addition, the impact on the body during landings must be attenuated in the lower extremity joints. The purpose of this study was to investigate landing biomechanics during landing with dynamic knee valgus by measuring the vertical ground reaction force (vGRF) and angular impulses in the lower extremity during a single-leg landing. The study included 34 female college students, who performed the single-leg drop vertical jump. Lower extremity kinetic and kinematic data were obtained from a 3D motion analysis system. Participants were divided into valgus (N = 19) and varus (N = 15) groups according to the knee angular displacement during landings. The vGRF and angular impulses of the hip, knee, and ankle were calculated by integrating the vGRF-time curve and each joint’s moment-time curve. vGRF impulses did not differ between two groups. Hip angular impulse in the valgus group was significantly smaller than that in the varus group (0.019 ± 0.033 vs. 0.067 ± 0.029 Nms/kgm, p<0.01), whereas knee angular impulse was significantly greater (0.093 ± 0.032 vs. 0.045 ± 0.040 Nms/kgm, p<0.01). There was no difference in ankle angular impulse between the groups. Our results indicate that dynamic knee valgus increases the impact the knee joint needs to attenuate during landing; conversely, the knee varus participants were able to absorb more of the landing impact with the hip joint.

## Introduction

The anterior cruciate ligament (ACL) is frequently injured during soccer, basketball, and many other sports. In recent studies, the mechanism of ACL injury has been widely considered to involve biomechanical factors [1–3]. An increased knee valgus angle during landings is one of the main causative factors for knee injuries, including injuries to the ACL [4–6]. Dynamic knee valgus, described as a combination of hip adduction, hip internal rotation, and knee abduction is recognized as a common lower extremity alignment seen in non-contact ACL injury situations [4,7,8]. Prospective studies have reported that increased knee valgus angle and knee abduction moment during landings were predictive of non-contact ACL injuries in female athletes [4,9,10]. These studies suggested the importance of knee injury prevention for athletes who land with dynamic knee valgus.

Previous studies have suggested that a diminished capacity to attenuate the impact imposed on the body during the deceleration phase of landings is one of the factors that cause knee injuries, including injuries to the ACL [11,12]. To prevent knee injury, the impact imposed on the body must be attenuated in the lower extremity joints to create soft landings. Some researchers have measured the vertical ground reaction force (vGRF) and angular impulses in the lower extremity joints to evaluate lower extremity biomechanics in landings [6,13]. DeVita and Skelly reported that soft landings, defined as a knee flexion angle greater than 90 degrees after landing from a vertical height of 59 cm, resulted in a lower vGRF and ankle plantar flexor impulse than did stiff landings [14]. Thus, soft landings are effective in reducing the impact applied to the lower extremity joints by the ground during the ground contact phase. From these findings, lower extremity kinetic variables, including the vGRF and angular impulses, may be useful parameters for evaluating soft landings. Recent reports have suggested that landing with knee valgus alignment may result in poor dynamic lower extremity alignment, such as dynamic knee valgus, which is described as the result of a combination of hip adduction, hip internal rotation, and knee abduction that results in increased knee valgus or increased knee abduction moment [4,7,8]. Previous studies have suggested that a diminished capacity to attenuate the impact imposed on the body during landings is one of the factors to cause knee injuries [11,12]. Additionally, altered knee dynamic alignment may reduce the capacity to attenuate the impact imposed on the lower extremity during the deceleration phase of landings. However, reports concerning the characteristics of lower extremity kinetics during landings with dynamic knee valgus are limited. Additionally, there has been no consideration of how landing with dynamic knee valgus affects the lower extremity kinetics, such as vGRF and joint angular impulses, reportedly related to soft landings. The purpose of this study was to investigate the characteristics of lower extremity biomechanics during landing with dynamic knee valgus by measuring the vGRF and joint angular impulses in the lower extremities during the deceleration phase of a single-leg landing. Our hypothesis was that subjects who land with dynamic knee valgus would exhibit greater vGRF and joint angular impulses in the lower extremities, indicative of a stiff landing, compared to those who land with dynamic knee varus.

## Methods

### Participants

Thirty-four female college students (age: 20.7±1.8 years old; height: 159.9±5.6 cm; weight: 52.7±5.9 kg) volunteered to participate in this study. For inclusion, participants were required to have no history of orthopedic hip, knee, or ankle surgery. The dominant foot, determined according to which foot was used to kick a ball [15], was the right foot in 30 subjects and the left foot in 4 subjects. All participants gave written informed consent for participation prior to testing and were then shown the testing sequence by assistant researchers. This study followed the Declaration of Helsinki and was approved by the Ethics Committee at the Saitama Medical University, Saitama, Japan (M-54). The participants mentioned in this manuscript have also given their written informed consent to the publication of these case details.

### Instrumentation

A three-dimensional motion analysis system with eight cameras (Vicon MX System, Vicon Motion Systems, Oxford, UK) was used to record lower extremity kinematic and kinetic data during the single-leg drop vertical jump. Kinematic and kinetic data were sampled at 240 Hz and low pass filtered at 16 Hz with a fourth-order zero lag Butterworth filter. Thirty-five reflective markers were placed on specific anatomical landmarks (left and right sides of the front of the head, left and right sides of the back of the head, 7th cervical vertebra, 10th thoracic vertebra, clavicle, sternum, right back, shoulders, lateral epicondyles of the elbows, medial wrists, lateral wrists, second metacarpal heads, anterior superior iliac spines, posterior superior iliac spines, lateral thighs, lateral epicondyles of the knees, lateral tibias, lateral malleolus, second metatarsal heads, and heels). Vicon’s Plug-in-Gait full body model (Vicon Motion Systems, Oxford, UK) was used to derive the lower extremity kinematic data [16]. Two force plates (MSA-6 Mini Amp, AMTI, MA, USA) were used to record vGRF during the landing phase of a single-leg drop vertical jump. The vGRF sampling rate was set at 1200Hz.

### Experimental procedures

**Fig 1:**
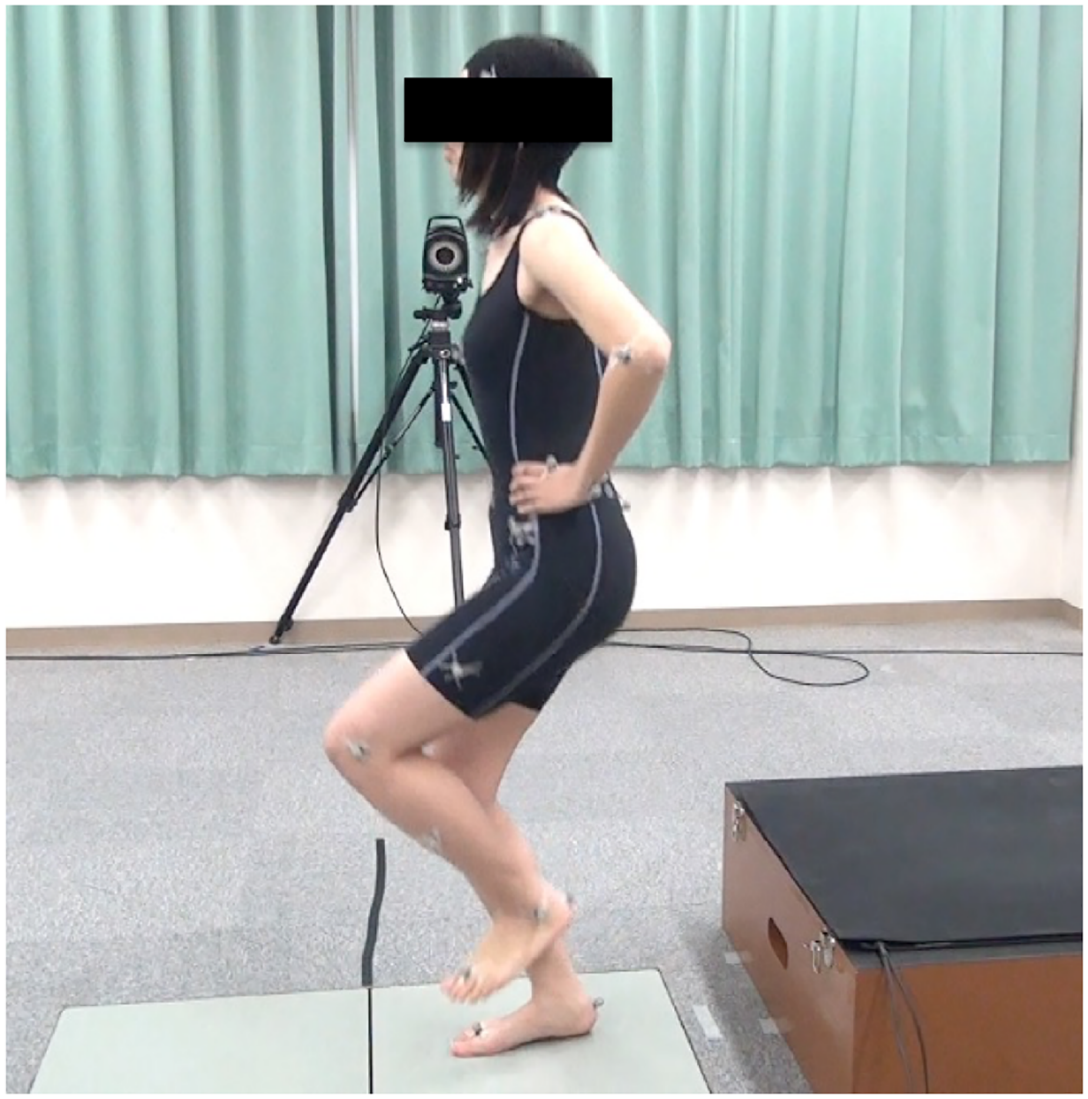
The landing phase of a single-leg drop vertical jump. A single-leg drop vertical jump on their dominant foot consisted of two stages: first, landing after dropping down from a 40 cm box; and second, landing after a maximal vertical jump rebounding from first drop.

The participants wore close fitting dark shorts to aid data collection. They performed a single-leg drop vertical jump on their dominant foot (Fig 1). This jump consisted of two stages: first, landing after dropping down from a 40 cm box; and second, landing after a maximal vertical jump rebounding from the first drop. All participants were shown the testing sequence by assistant researchers. Several practice trials were conducted to enable the participants to perform a single-leg drop vertical jump in the correct fashion. Subsequent trials were repeated until data from five successful trials have been collected. Trials were excluded if the subject lost her balance during the landing process.

### Data collection

All kinematic and kinetic data were calculated during the deceleration phase of the single-leg drop vertical jumps, defined as the period from initial ground contact to the moment when the greatest vGRF was recorded during the first landing. The initial ground contact was defined as the point when the vGRF reached more than 10 N. All variables were calculated as the mean of the middle three trials out of five successful trials.

The knee valgus or varus angle of each participant was calculated from the filtered three-dimensional coordinate data. Participants were divided into two groups, the valgus group (N = 19, valgus angle 4.4 ± 3.0°) and the varus group (N = 15, valgus angle - 5.3 ± 4.0°). Knee angular displacement was calculated by subtracting the angle exhibited at initial ground contact from the angle exhibited at the time of peak vGRF.

Peak vGRF was the maximum value recorded during the landing phase and normalized to the subject’s body weight (kg). The peak vGRF represents the point of changing from a landing to a jump during the landing. Angles and moments of the lower extremity joints in the sagittal plane were recorded at two points, when the vGRF and their respective values were maximized during the landing phase. Joint moments were normalized to the product of the subject’s body weight (kg) and height (m). In addition, the time (ms) from initial ground contact to peak vGRF was recorded.

The vGRF impulses (Ns/kg) were calculated by integrating the vGRF-time curve over the deceleration phase of the landing, and then normalizing this according to the subject’s body weight (kg). Hip, knee, and ankle angular impulses were calculated by integrating the respective joint moment-time curves during the deceleration phase of the landings. Further, these were normalized according to the product of the subject’s body weight (kg) and height (m). All variables were calculated as the mean of the middle three trials from out of five successful trials.

### Statistical analysis

Data were analyzed using SPSS version 19.0 (SPSS, Chicago, IL). Unpaired t tests were used to compare changes in the joint angles, moments, vGRF, vGRF impulse, and joint angular impulses between the valgus and varus groups. The joint angles and moments were compared between the two groups at peak vGRF and their respective peak values were recorded during the landing phase. Significance was set at a level of *p* < 0.05.

## Results

### Knee valgus angle of the knee valgus and varus groups

Values for the knee valgus angle of the valgus and varus groups were 4.4 ± 3.0° and - 5.3 ± 4.0°, respectively. There was a significant difference in knee valgus angles between the valgus and varus groups (p<0.001).

### The vGRF and vGRF impulse

**Fig 2:**
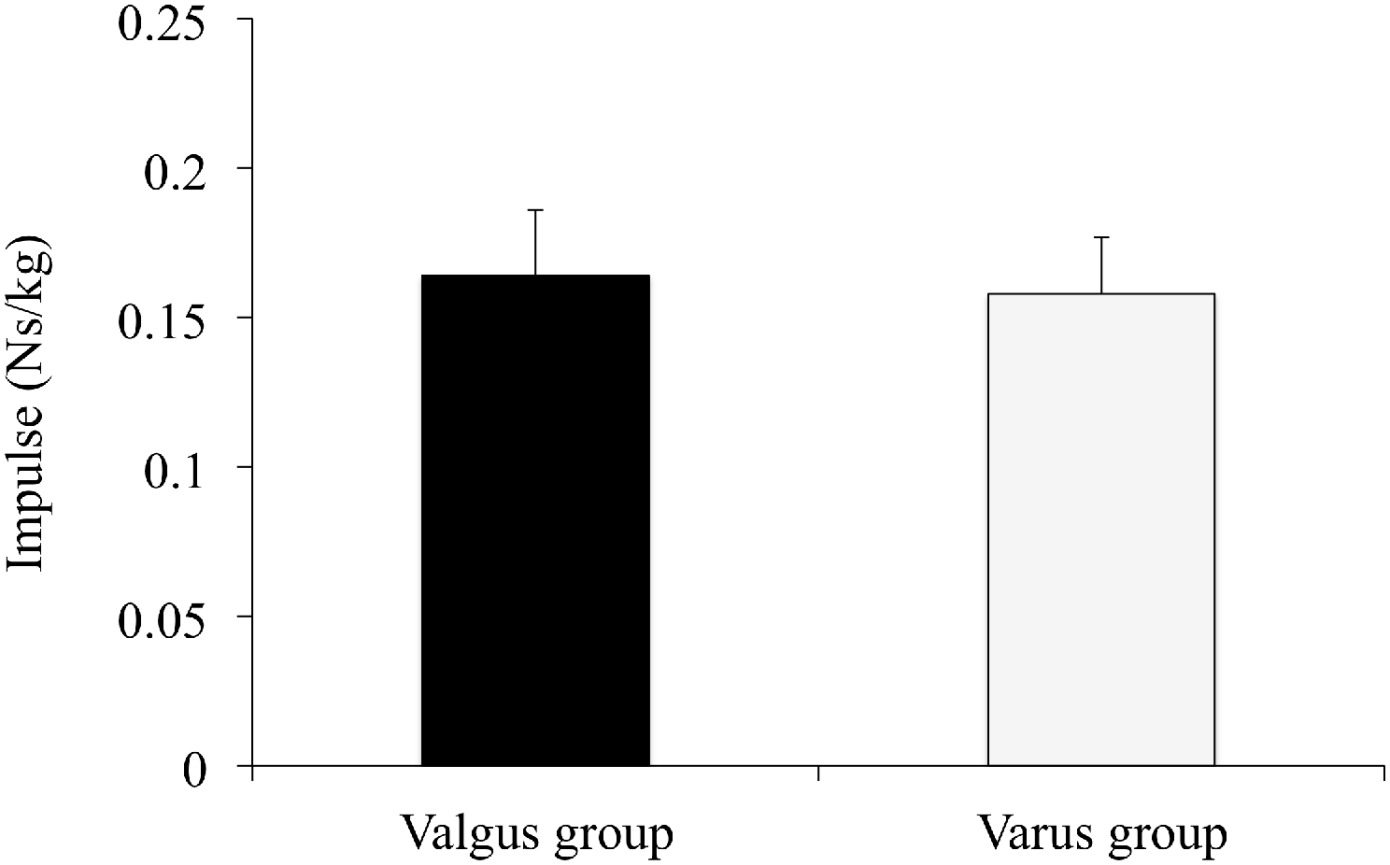
The vGRF impulse of the knee valgus and varus groups. This figure represents the means of the vGRF impulse and standard deviation of the knee valgus and varus groups during the deceleration phase of a single-leg drop vertical jump. These variables were normalized to the subject’s body weight (kg).

Values for the peak vGRF and vGRF impulse in the valgus and varus groups are shown in Table 1 and Fig 2. Neither vGRF impulse (0.164 ± 0.022 vs. 0.158 ± 0.019 Ns/kg, p = 0.41) nor peak vGRF (3.63 ± 0.52 vs. 3.43 ± 0.49 N/kg, p = 0.26) during the landing phase differed between the two groups. The time (ms) from initial ground contact to peak vGRF was 61.2 ± 9.32 ms (14.9 ± 4.9% to the landing phase) in the valgus group and 69.4 ± 19.4 ms (16.1 ± 8.1% to the landing phase) in the varus group; this difference was not statistically significant (p = 0.64).

**Table 1:** Comparisons of the vGRF and lower extremity kinetics between the valgus and varus groups.

	Valgus Group ^a^ N = 19	Varus Group ^a^ N = 15	p value
Peak vGRF (N/kg)	3.63 ± 0.52	3.43 ± 0.49	0.26
Hip Joint			
Peak extensor moment (Nm/kgm)	3.29 ± 1.86	3.78 ± 1.18	0.38
Extensor moment at peak vGRF (Nm/kgm)	2.43 ± 1.54	3.18 ± 1.15	0.12
Knee Joint			
Peak extensor moment (Nm/kgm)	3.10 ± 0.71	2.33 ± 0.86	0.01 ^b^
Extensor moment at peak vGRF (Nm/kgm)	1.82 ± 0.86	1.14 ± 0.98	0.04 ^c^
Ankle Joint			
Peak plantar flexor moment (Nm/kgm)	3.38 ± 0.77	3.74 ± 0.88	0.21
Plantar flexor moment at peak vGRF (Nm/kgm)	3.05 ± 0.91	3.48 ± 0.94	0.19

^a^ Data are reported as mean ± standard diviation.

^b^ Statistically significant at p < 0.01.

^c^ Statistically significant at p < 0.05.

vGRF, vertical ground reaction force

### Angular impulses in the lower extremity joints

**Fig 3:**
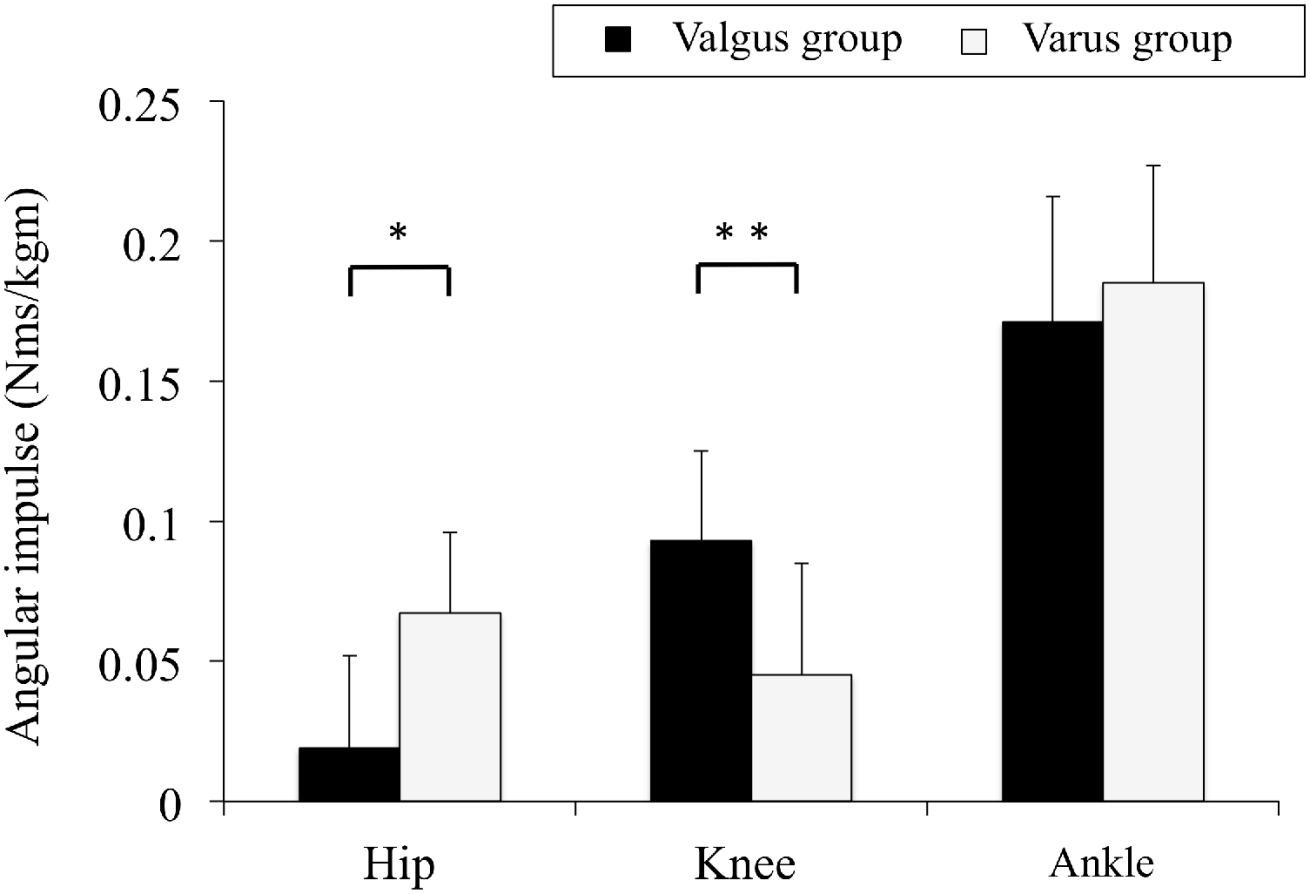
Angular impulses in the lower extremity joints of the knee valgus and varus groups. This figure represents the means of the hip, knee, and ankle angular impulses and standard deviations of the knee valgus and varus groups during the deceleration phase of a single-leg drop vertical jump. These variables were normalized to the product of the subject’s body weight (kg) and height (m). Asterisks indicate significant differences between the valgus and varus groups (*; p<0.01, **; p<0.05).

Values for hip, knee, and ankle angular impulses in the valgus and varus groups are shown in Fig 3. Hip angular impulse in the valgus group was significantly lower than that in the varus group (0.019 ± 0.033 vs. 0.067 ± 0.029 Nms/kgm, p = 0.01), whereas knee angular impulse was significantly greater (0.093 ± 0.032 vs. 0.045 ± 0.040 Nms/kgm, p = 0.01). There was no difference between the two groups in ankle angular impulse (0.171 ± 0.045 vs. 0.185 ± 0.042 Nms/kgm, p = 0.35).

### Moments and angles in the lower extremity joints

**Fig 4:**
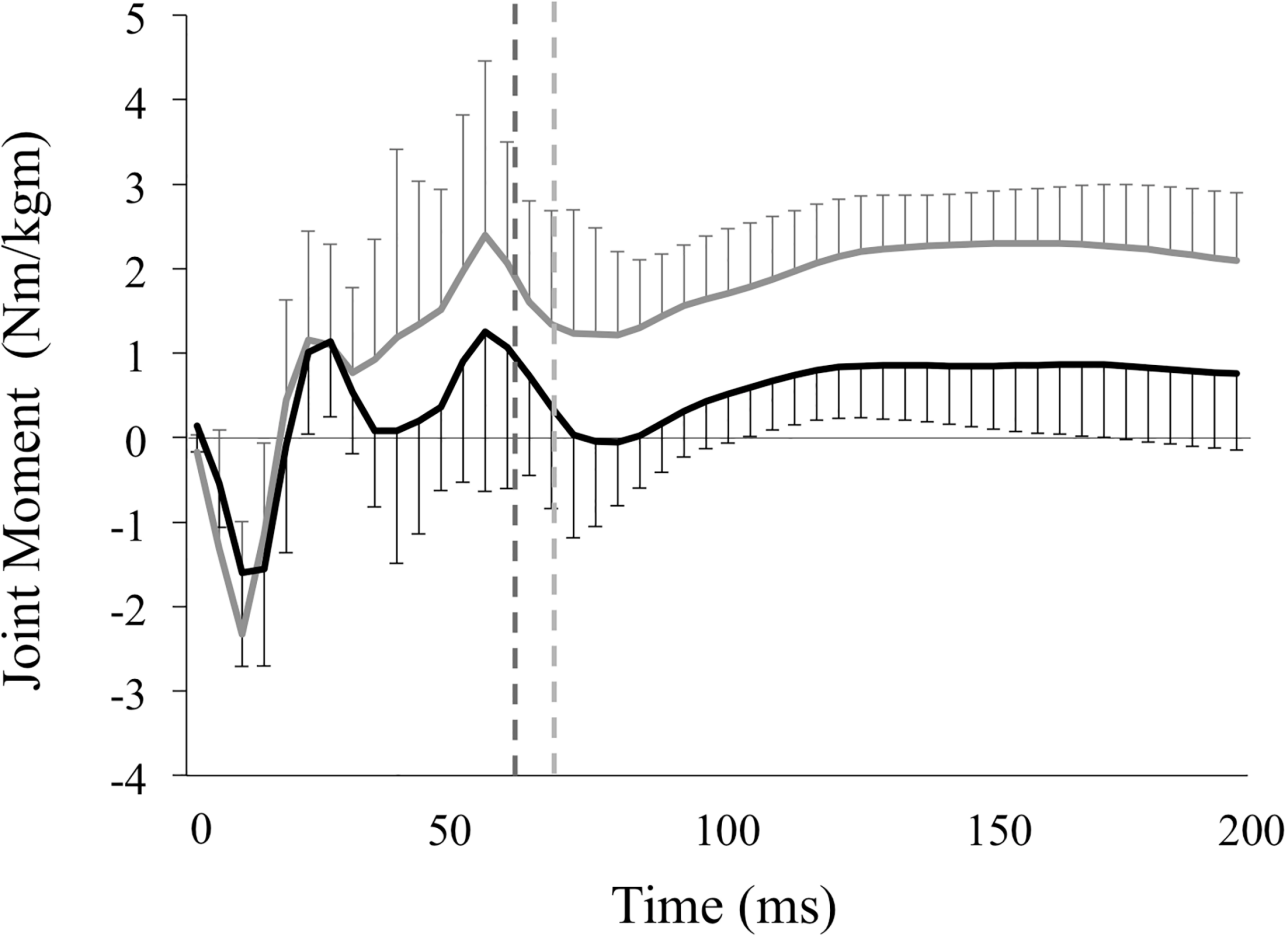
Moments in the hip joint of the knee valgus and varus groups. This figure represents the means of the hip extensor moment and standard deviations of the knee valgus (solid black lines) and knee varus groups (solid gray lines) during the landing phase. These variables were normalized to the product of the subject’s body weight (kg) and height (m). The vertical broken lines represent the end of the deceleration phase, which is defined as the period from the initial ground contact to the peak of vGRF in the valgus (black lines, 61.2 ± 9.32 ms) and varus groups (gray lines, 69.4 ± 19.4 ms). Variables were calculated by integrating the hip extensor moment—time curves during the deceleration phase, representing the hip extensor angular impulse.

**Fig 5:**
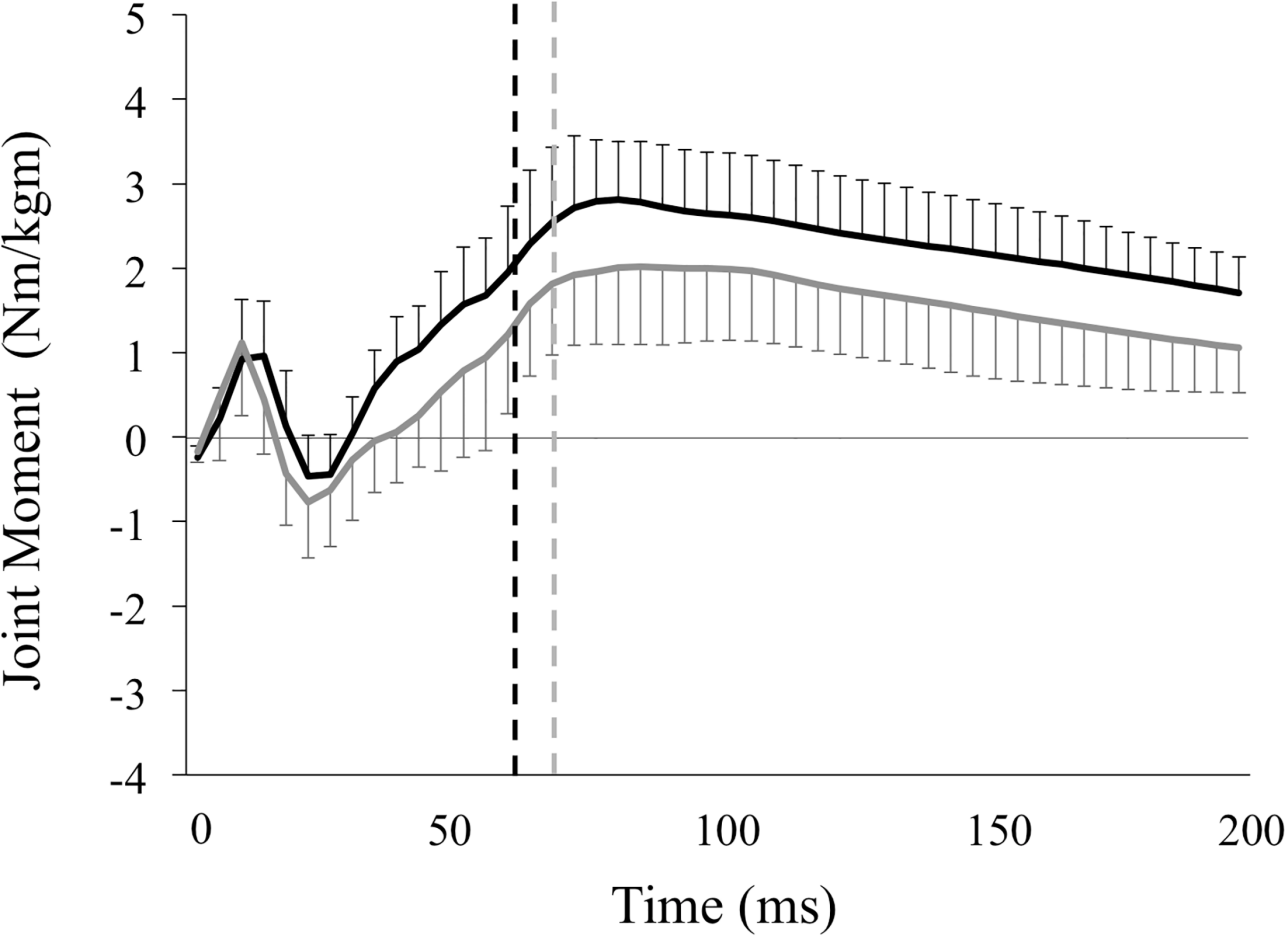
Moments in the knee joint of the knee valgus and varus groups. This figure represents the means of the knee extensor moment and standard deviations of the knee valgus (solid black lines) and knee varus groups (solid gray lines) during the landing phase. These variables were normalized to the product of the subject’s body weight (kg) and height (m). The vertical broken lines represent the end of the deceleration phase, which is defined as the period from the initial ground contact to the peak of vGRF in the valgus (black lines, 61.2 ± 9.32 ms) and varus groups (gray lines, 69.4 ± 19.4 ms). Variables were calculated by integrating the knee extensor moment—time curves during the deceleration phase, representing the knee extensor impulse.

**Fig 6:**
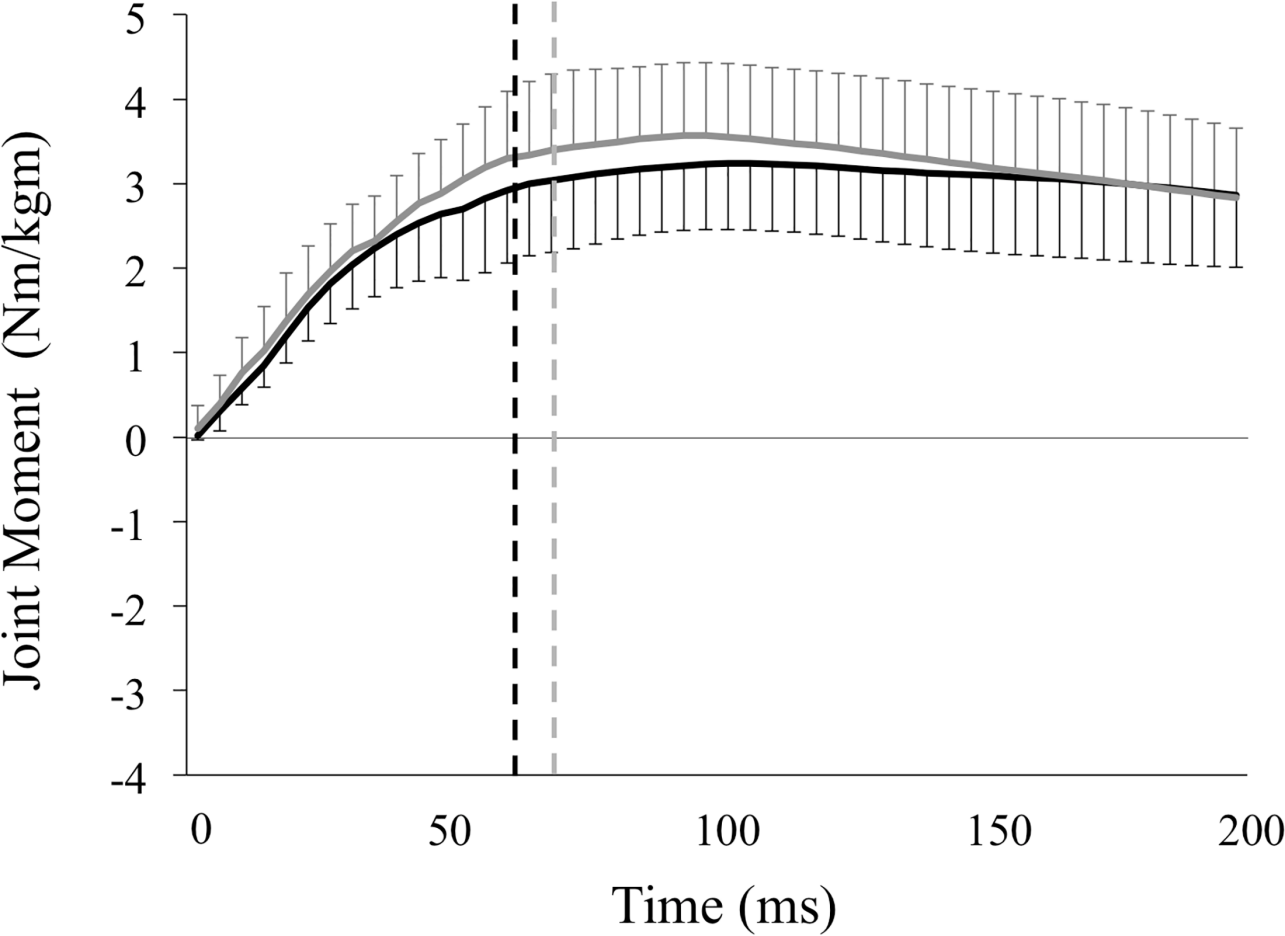
Moments in the ankle joint of the knee valgus and varus groups. This figure represents the means of the ankle plantar flexor moment and standard deviations of the knee valgus (solid black lines) and knee varus groups (solid gray lines) during the landing phase. These variables were normalized to the product of the subject’s body weight (kg) and height (m). The vertical broken lines represent the end of the deceleration phase, which is defined as the period from the initial ground contact to the peak of vGRF in the valgus (black lines, 61.2 ± 9.32 ms) and varus groups (gray lines, 69.4 ± 19.4 ms). Variables were calculated by integrating the ankle plantar flexor moment—time curves during the deceleration phase, representing the ankle plantar flexor angular impulses.

Values for hip, knee extensor, and angle plantar flexor moments in the valgus and varus groups are shown in Table 1 and Figs 4–6. The peak knee extensor moment was significantly greater in the valgus group than in the varus group (3.10 ± 0.71 vs. 2.33 ± 0.86 Nm/kgm, p = 0.01), as was the knee extensor moment at peak vGRF (1.82 ± 0.86 vs. 1.14 ± 0.98 Nm/kgm, p = 0.04). In contrast, peak hip extensor moment (3.29 ± 1.86 vs. 3.78 ± 1.18 Nm/kgm, p = 0.38) and ankle plantar flexor moment (3.38 ± 0.77 vs. 3.74 ± 0.88 Nm/kgm, p = 0.21) did not differ between the groups. There was also no significant difference between the two groups in hip extensor moment (2.43 ± 1.54 vs. 3.18 ± 1.15 Nm/kgm, p = 0.12) or ankle plantar flexor moment at peak vGRF (3.05 ± 0.91 vs. 3.48 ± 0.94 Nm/kgm, p = 0.19).

Values for hip flexion, knee flexion, and ankle dorsi-flexion angles in the valgus and varus groups are shown in Table 2. Peak hip flexion (40.2 ± 10.2 vs. 44.4 ± 11.7°, p = 0.27), knee flexion (58.1 ± 6.8 vs. 60.4 ± 7.8°, p = 0.36) and ankle dorsi-flexion angles (29.3 ± 4.4 vs. 31.9 ± 7.6°, p = 0.22) did not significantly differ between the two groups. Similarly, there was no significant difference between the two groups in hip flexion (29.5 ± 7.8 vs. 31.3 ± 7.6°, p = 0.27), knee flexion (37.1 ± 4.9 vs. 36.6 ± 7.1°, p = 0.36), or ankle dorsi-flexion angles (15.9 ± 4.8 vs. 15.7 ± 4.2°, p = 0.22) at peak vGRF.

**Table 2:** Comparisons of lower extremity kinematics between the valgus and varus groups.

	Valgus Group ^a^ N = 19	Varus Group ^a^ N = 15	P value
Hip Joint			
Peak flexion angle (deg)	40.2 ± 10.2	44.4 ± 11.7	0.27
Flexion angle at peak vGRF (deg)	29.5 ± 7.8	31.3 ± 7.6	0.50
Knee Joint			
Peak flexion angle (deg)	58.1 ± 6.8	60.4 ± 7.8	0.36
Flexion angle at peak vGRF (deg)	37.1 ± 4.9	36.6 ± 7.1	0.80
Ankle Joint			
Peak dorsi-flexion angle (deg)	29.3 ± 4.4	31.9 ± 7.6	0.22
Dorsi-flexion angle at peak vGRF (deg)	15.9 ± 4.8	15.7 ± 4.2	0.89

^a^ Data are reported as mean ± standard diviation.

vGRF, vertical ground reaction force

## Discussion

The vGRF and angular impulses in the lower extremity joints explain the net force and joint moments experienced by the lower extremity joints over periods of time during the deceleration phase of landings. From the results of our study, the subjects with dynamic knee valgus experienced greater knee angular impulse compared with those with dynamic knee varus. These findings indicate that landing with dynamic knee valgus may increase the impact on the knee joint during the deceleration phase of landings. Therefore, dynamic knee valgus during landings may be one of the biomechanical factors that reduce an individual’s capacity to attenuate the impact imposed on the knee joint during landings. Some researchers have reported that the knee and hip joints are the primary shock absorber during landings [12,17,18]. In addition, it has been shown in females that the hip extensor, knee extensor, and ankle plantar flexor muscles contribute 38, 41, and 22% of the total energy absorption, respectively [19]. Some of these reports have indicated that the knee joint is the most important impact absorber of the lower extremities [12,17,20]. The results of the present study show that the hip angular impulse in dynamic knee valgus is smaller than that in dynamic knee varus. Therefore, the knee joint was the most important impact absorber in the participants who landed with dynamic knee valgus, while the impact absorption by the hip joint was small. In addition, the vGRF impulse represents the total impact applied to the body, including the hip, knee, and ankle joints, by the ground during landings. In this study, vGRF impulse differed between the two knee alignment groups in the frontal plane. These results indicate that the knee angular impulse in the valgus group was apparently distributed to the hip joints without changing the total impact imposed on the body during landings.

Peak knee extensor moment in the subjects with dynamic knee valgus was greater than in those with dynamic knee varus. The knee extensor moment, defined as internal joint moment, refers to the moment of the force that tends to rotate the knee joint in the direction of the sagittal plane [21]. In addition, it is generated by active knee extensor muscles such as the rectus femoris and vastus medialis oblique. Accordingly, the result of this study indicate that landing on with dynamic knee valgus increase the knee extension moment during the landing phase, i.e., there was greater activation of the knee extensor muscles in the subjects with dynamic knee valgus than in those with dynamic knee varus.

During the landing phase, the knee flexion angle was not affected by the difference in knee dynamic alignment in the frontal plane. Similarly, the hip flexion and ankle dorsi-flexion angles did not significantly differ between groups. These results show that landings with dynamic knee valgus increase the knee extensor angular impulse and extensor moment applied to the knee joint, irrespective of the dynamic knee alignment in the sagittal plane during the landing phase.

In terms of impact attenuation during landings, these findings indicate that altering the landing strategy in motor learning could change the load on the hip and knee joints. In addition, previous studies have suggested that a diminished capacity to attenuate the impact on the body during landings might cause knee injuries [11,12]. This indicates that increased impact during landings is one of the factors responsible for an increase in mechanical stress in the knee joint. Further, altering the landing strategy with respect to the knee joint could be useful when considering movement strategies for landings by athletes with knee injuries such as ACL tears.

This study had a potential limitation, in that trials of single-leg drop vertical jumps were excluded from the analysis if participants could not successfully complete the landing. Failed trials included those when the subject lost her balance during the landing process. However, failed trials may involve a different landing mechanism than successful trials, related to the impact absorption by the lower extremities. Some researchers have reported that neuromuscular control and landing strategy during landings differs between successful and failed trials due to earlier muscle onset and greater amplitude [22]. For this reason, future studies should take into account failed trials and analyze the differences between successful and failed trials with dynamic knee valgus.

## Conclusion

The impact on the body during the deceleration phase of landings needs to be attenuate in the lower extremity joints to create soft landings. Our study showed that landing with dynamic knee valgus resulted in a greater knee extensor angular impulse than landing with dynamic knee varus, whereas the hip extensor angular impulse was smaller. The hip, knee, and ankle angles were not affected by the difference in dynamic knee alignment in the frontal plane. These results suggest that landing with dynamic knee valgus may reduce the capacity to attenuate the impact imposed on the knee joint during the deceleration phase of landings, without changing the dynamic knee alignment in the sagittal plane. In addition, the impact on the knee joint was apparently counteracted by the capacity of the hip joint for impact absorption. These findings indicate that altering the landing strategy in motor learning could change the load on the hip and knee joints in terms of impact attenuation during landings.

## Supporting information

S1 File The lower extremity joint angles, moments, angular impulses and vGRF parameters of each subject during landings. (XLSX)
